# Evolution from a respiratory ancestor to fill syntrophic and fermentative niches: comparative fenomics of six *Geobacteraceae *species

**DOI:** 10.1186/1471-2164-10-103

**Published:** 2009-03-11

**Authors:** Jessica E Butler, Nelson D Young, Derek R Lovley

**Affiliations:** 1 Department of Microbiology, 203 Morrill Science Center IVN, University of Massachusetts, 639 North Pleasant Street, Amherst, MA 01003, USA

## Abstract

**Background:**

The anaerobic degradation of organic matter in natural environments, and the biotechnical use of anaerobes in energy production and remediation of subsurface environments, both require the cooperative activity of a diversity of microorganisms in different metabolic niches. The *Geobacteraceae *family contains members with three important anaerobic metabolisms: fermentation, syntrophic degradation of fermentation intermediates, and anaerobic respiration.

**Results:**

In order to learn more about the evolution of anaerobic microbial communities, the genome sequences of six *Geobacteraceae *species were analyzed. The results indicate that the last common *Geobacteraceae *ancestor contained sufficient genes for anaerobic respiration, completely oxidizing organic compounds with the reduction of external electron acceptors, features that are still retained in modern *Geobacter *and *Desulfuromonas *species. Evolution of specialization for fermentative growth arose twice, via distinct lateral gene transfer events, in *Pelobacter carbinolicus *and *Pelobacter propionicus*. Furthermore, *P. carbinolicus *gained hydrogenase genes and genes for ferredoxin reduction that appear to permit syntrophic growth via hydrogen production. The gain of new physiological capabilities in the *Pelobacter *species were accompanied by the loss of several key genes necessary for the complete oxidation of organic compounds and the genes for the *c*-type cytochromes required for extracellular electron transfer.

**Conclusion:**

The results suggest that *Pelobacter *species evolved parallel strategies to enhance their ability to compete in environments in which electron acceptors for anaerobic respiration were limiting. More generally, these results demonstrate how relatively few gene changes can dramatically transform metabolic capabilities and expand the range of environments in which microorganisms can compete.

## Background

The global carbon cycle and the production of a number of biofuels depends on the cooperative interaction of a physiological diversity of anaerobic microorganisms. These include microorganisms that ferment complex substrates to simpler molecules; respiratory microorganisms that convert fermentation products to carbon dioxide and/or methane; and syntrophic organisms that anaerobically oxidize some fermentation products and other substrates in reactions that are only thermodynamically feasible when respiratory organisms consume the syntrophs' products. The family *Geobacteraceae *has representatives of all three metabolic groups, providing an opportunity to evaluate how microorganisms might evolve to fill these various niches in anaerobic ecosystems.

Within the *Geobacteraceae*, *Geobacter *and *Desulfuromonas *are specialists in the complete oxidation of organic compounds to carbon dioxide coupled to the reduction of insoluble, extracellular electron acceptors [1]. In aquatic sediments and submerged soils they influence the cycling of carbon and metals by oxidizing acetate, the central fermentation intermediate in anaerobic environments, with the reduction of iron and manganese oxides [1,2]. In hydrocarbon-contaminated subsurface environments *Geobacter *species can play an important role in the bioremediation of aromatic hydrocarbons by oxidizing the contaminants with the reduction of Fe(III) oxides naturally present in the subsurface [3,4]. This is a process which can be artificially stimulated [5,6]. Another bioremediation application involving *Geobacter *species is to add acetate to uranium-contaminated subsurface environments. This stimulates the growth of *Geobacter *species which reduce soluble, mobile U(VI) to insoluble U(IV) [7] and thus immobilize the uranium *in situ *[4,8]. The ability of *Geobacter *and *Desulfuromonas *species to oxidize organic compounds with electron transfer to graphite electrodes provides a convenient method for harvesting electricity from aquatic sediments and organic wastes to power electronic devices [9-11].

In contrast, the primary niche of *Pelobacter *species is methanogenic environments, functioning either as fermentative microorganisms or living in syntrophic associations with methanogens by partially oxidizing organic compounds to hydrogen and acetate which the methanogens consume [12,13]. *Pelobacter *species cannot oxidize acetate or other organic compounds completely to carbon dioxide and are ineffective in electron transfer to metals [14] or electrodes [15]. Therefore, not only the natural environments, but also the technological applications of *Geobacter/Desulfuromonas *versus *Pelobacter *species are quite different.

Here we report on the sequenced genomes of six members of the *Geobacteraceae *family: *Geobacter sulfurreducens *[16], *Geobacter metallireducens*, *Geobacter uraniireducens*, *Pelobacter propionicus*, *Pelobacter carbinolicus*, and *Desulfuromonas acetoxidans*. We use comparative genomics in an effort to better understand the enzymes involved in these unusual metabolisms, and to provide insight in to the selective pressures that may have contributed to their evolution.

## Results and discussion

### Identification of protein families in the six genomes

The general features of each of the six genomes are presented in Table 1. Orthologous proteins, those predicted to have similar functions in the different organisms, were identified by Markov clustering of sets of reciprocal best BLAST matches [17]. Using all 21,716 protein coding genes in the six genomes (see Additional file 1), 3,696 clusters with members from at least two genomes were defined (see Additional file 2). 15,207 proteins (70.0%) were classified into clusters. A functional role was predicted for each cluster using the *G. sulfurreducens in silico *model annotation [18] and COG categorization [19].

**Table 1:** General features of the *Geobacteraceae *genomes

	*Geobacter sulfurreducens*	*Geobacter metallireducens*	*Geobacter uraniireducens*	*Pelobacter propionicus*	*Pelobacter carbinolicus*	*Desulfuromonas acetoxidans*
	
**NCBI ID**	NC_002939	NC_007517	NC_009483	NC_008609	NC_007498	NZ_AAEW00000000
**Contigs**	1	1	1	1	1	51
**Length (nt)**	3,814,139	3,997,420	5,136,364	4,008,000	3,665,893	3,828,328
**GC Content (%)**	60	59	54	59	55	51
**Protein coding**	3446	3519	4357	3576	3352	3234
**rRNA operons**	2	2	2	4	2	1
**Plasmids**	none	13.8 kb	none	30.7 kb and 202.4 kb	none	n/a

The pattern of conservation of the proteins families across all species (the phyletic pattern) was determined (see Additional files 2 and 3). By far the most common pattern was conservation across all species, 5,345 proteins were members of clusters that included at least one protein from each genome (see Additional file 3). The second most common pattern was conservation in the *Geobacter *species and *P. propionicus *only (1,158 proteins). Proteins unique to the respiratory or fermentative species were relatively rare. Only 370 proteins were found in the respiratory species but not the fermentative species (see Additional file 4), and only 260 proteins were found in the fermentative species but not the respiratory species (see Additional file 5).

### Whole-genome phylogeny

**Figure 1:**
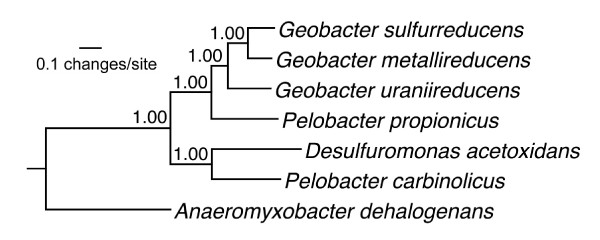
**Genome-based *Geobacteraceae *phylogeny**. Bayesian inference of the phylogenetic tree of the six *Geobacteraceae *species discussed in the text, using another *Deltaproteobacterial *species, *Anaeromyxobacter dehalogenans*, as the outgroup. The tree was based on a concatenation of the proteins in the 481 families that had exactly one ortholog conserved in each of the seven genomes (see Additional file 6). Values at branch points are posterior probabilities.

A phylogeny of the family was constructed using the 481 protein families that had a single protein from each of the six genomes and the outgroup species *Anaeromyxobacter dehalogenans *(see Additional file 6). These proteins had diverse functions, including information storage, metabolism, cell signaling, and unknown. The proteins from each genome were concatenated then aligned, and this alignment was used to create a Bayseian model of the phylogeny (Figure 1). This analysis confirmed that, as housekeeping-gene phylogeny suggests [20], the *Geobacteraceae *can be divided into two clades, and that the two fermentative *Pelobacter *species are not most closely related to each other, instead there is one *Pelobacter *species in each clade of the respiratory species (Figure 1).

Thus, based on phylogeny, there are at least three possibilities for how modern *Geobacteraceae *evolved to fill multiple metabolic niches. The ancestor may have been adapted to a fermentative/syntrophic lifestyle with no reliance on external electron acceptors. In this scenario, the *Geobacter *and *Desulfuromonas *species would have independently evolved strategies for the complete oxidation of organic compounds coupled to Fe(III) reduction. Alternatively, the ancestor may have been a respiratory microorganism, and the *Pelobacter *species independently evolved strategies for fermentation and syntrophy and lost the ability to transfer electrons out of the cell. Another option was that the ancestor may have had both types of metabolism, and the various branches of the family lost certain abilities. We investigated these possibilities by analyzing the conservation and evolutionary history of genes for energy metabolism.

### Conservation of pathways for acetate oxidation

**Figure 2:**
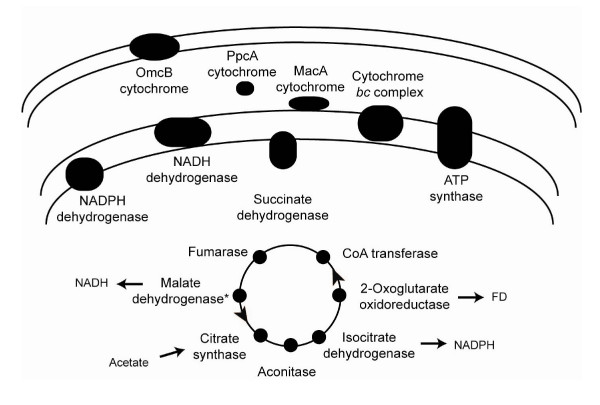
**The pathways of energy metabolism conserved in the respiratory *Geobacteraceae *species**. Shown are the pathways, based those characterized in *G. sulfurreducens*, for acetate activation and oxidation via the TCA cycle in the cytoplasm, for inner membrane oxidation of TCA cycle products coupled with electron and proton transport, and for ATP generation. The genes encoding the enzymes of these pathways and their conservation pattern in all of the *Geobacteraceae *genomes are given in see Additional file 7. The enzymes are colored black if there were orthologs for every subunit in all of the respiratory species. The asterisk indicates that malate dehydrogenase activity has been shown in *D. acetoxidans*, though genes for the enzyme were not found in the draft sequence of the genome. The pathway for electron transfer through the periplasm and out to the external electron acceptor is not well characterized in these species, and is represented here by the three cytochromes known to be required *in vivo *in *G*. *sulfurreducens *that are also conserved in all of the respiratory species.

In the respiratory species *G. sulfurreducens*, *G. metallireducens*, *G. uraniireducens*, and *D*. *acetoxidans*, acetate is the primary electron donor and it is oxidized via the TCA cycle, generating NADH, NADPH, and reduced ferredoxin (Figure 2) [21-23]. In contrast, *Pelobacter *species are incapable of acetate oxidation [12]. One protein family predicted to be acetate transporters (GSU0518) [24] was conserved in all of the acetate-oxidizing species (see Additional file 7). However, neither this protein nor any of the other acetate transporters found in *G. sulfurreducens *(GSU1068, GSU1070, and GSU2352) were conserved in either of the *Pelobacter *species (see Additional file 7). Twenty one genes encode the enzymes of the TCA cycle used by *G. sulfurreducens *[18] (Figure 2, see Additional file 7). There was full conservation in all of the respiratory species of at least one copy of each of these enzymes (see Additional file 7). The one exception was the malate dehydrogenase in *D. acetoxidans*. There was no gene predicted to encode a malate dehydrogenase in the incomplete version of the genome, but this enzyme activity is found in *D. acetoxidans *[23]. As with the acetate transporters, not all of the genetic redundancy seen in *G. sulfurreducens *in the enzymes of the TCA cycle was conserved across the family. Only one copy of the aconitase (GSU1660) and the keto/oxoacid ferredoxin oxidoreductase (GSU1859–GSU1862) were conserved in all the respiratory species (see Additional file 7).

The *Pelobacter *species contained orthologs to many of the TCA cycle enzymes (see Additional file 7), but there were two notable exceptions. NADPH metabolism appears to catalyzed by different enzymes in the *Pelobacter *species. A monomeric-type isocitrate dehydrogenase (GSU1465) was found in all of the respiratory species (see Additional file 8), but was not conserved in either *Pelobacter *species (see Additional file 7). In acetate oxidizers, this reaction is the primary source of NADPH in the cell [25]. Instead, the *Pelobacter *species contained non-orthologous isocitrate dehydrogenase genes (Ppro_0452 and Pcar_1038 in cluster 3107) of the more common, homodimeric type [25]. In addition, both *Pelobacter *species lacked the dehydrogenase (GSU0509–GSU0510) believed to transfer the electrons from NADPH into the electron transport chain [26]. This enzyme was conserved in all four of the respiratory species (see Additional file 7). These changes suggest that the NADPH produced by the *Pelobacter *species may not be used primarily as a source of electrons for energy metabolism, as it is for the respiratory species. Furthermore, *P. carbinolicus *contained a non-orthologous fumarase enzyme (Pcar_0324) predicted to be of the class II, Fe(III)-free type [27], rather than the class I, Fe(III) type found in the other *Geobacteraceae *(GSU0994).

### Conservation of pathways for extracellular electron transfer

**Figure 3:**
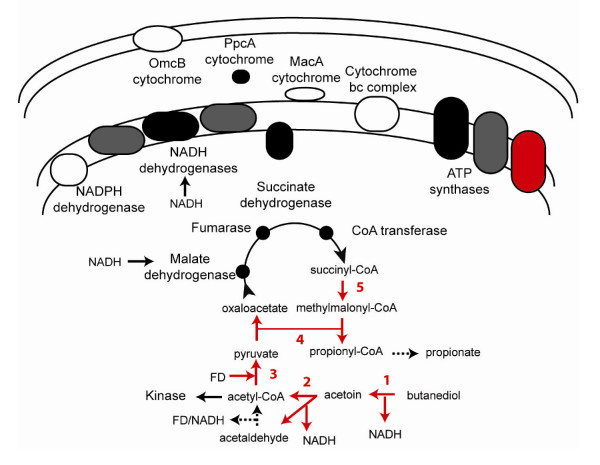
**The pathways of energy metabolism in *Pelobacter propionicus *and the differences from respiratory *Geobacteraceae *species**. Shown are the pathways of butanediol and acetoin fermentation used by *P. propionicus*. Vertically-inherited enzymes with orthologs in the other *Geobacteraceae *are shown in black. Both the vertically-inherited NADH dehydrogenase and ATP synthase have been duplicated in *P. propionicus *and these are shown in dark grey. Enzymes acquired by lateral gene transfer are shown in red: 1) butanediol dehydrogenase, 2) acetoin dehydrogenase, 3) pyruvate:ferredoxin oxidoreductase, 4) transcarboxylase, and 5) methylmalonyl-CoA mutase, and an ATP synthase. Enzymes conserved in the respiratory *Geobacteraceae *but lost in *P. propionicus *are shown in white. Reactions with multiple enzymes encoded in the genome for which there are diverse conservation patterns are shown as dashed arrows.

In the respiratory *Geobacteraceae *species, the reducing equivalents from the TCA cycle are passed to inner membrane quinones via NADH dehydrogenase (Figure 2) [21-23]. This is predicted to be the only step in the electron transport chain where protons are pumped across the inner membrane for ATP generation [18]. *G. sulfurreducens *encodes two NADH dehydrogenase operons, one with 12 subunits and one with 14 (see Additional file 7). The 14-subunit enzyme is conserved in all of the respiratory species and *P. propionicus *(see Additional file 7). In addition, *P. propionicus *appears to have recently duplicated this enzyme, there are three virtually identical copies in the genome (Ppro_0628–Ppro_0641, Ppro_1623–Ppro_1636, Ppro_3180–Ppro_3193). In contrast, *P. carbinolicus *lacks the 14-subunit enzyme, but encodes an ortholog to the 12-subunit enzyme (see Additional file 7). This conservation pattern indicates that the 14-subunit enzyme may be the more important for inner membrane proton and electron transport; it is conserved in the four respiratory species, and in the only fermentative species predicted to use an NADH dehydrogenase (as described below and in Figure 3).

The final part of the electron transport chain in the respiratory species is transfer of electrons from the inner membrane to cytochromes for transport to the cell surface. The differences between the respiratory and fermentative species are substantial in this part of the pathway. The genomes of all four of the respiratory species contained at least one copy of a putative cytochrome *bc *complex (GSU2932–GSU2934), which is predicted to transfer electrons from membrane-bound quinones to periplasmic cytochromes [28] (Figure 2, see Additional file 7). Neither of the *Pelobacter *species had any orthologs to this complex. *C*-type cytochromes span the periplasm and outer membrane in the respiratory species [1]. All of the *Geobacter *and *Desulfuromonas *genomes contained more than 100 ORFs that have the heme-binding motif [29] characteristic of *c*-type cytochromes (see Additional file 1). Both *Pelobacter *genomes had far fewer cytochrome genes, ca. 40 per genome (see Additional file 1). Many of the proteins found only in the respiratory species were multi-heme, *c*-type cytochromes (see Additional file 4), whereas none of the proteins specific to the fermentative species were multi-heme (see Additional file 5). In addition, both *Pelobacter *species lacked most of the specific cytochromes required for electron transfer in *G. sulfurreducens in vivo*. These include those conserved in all of the respiratory species: MacA, a *c*-type cytochrome associated with the inner membrane [30] and OmcB, an outer-membrane cytochrome [31].

### Lateral transfer of fermentation genes

Thus, while the *Pelobacter *species contained many vertically-inherited genes for respiratory metabolism, several key enzymes are different or missing in both species. To investigate evolution of the fermentative and syntrophic metabolism, *Pelobacter*-specific genes were analyzed, and genes originating from lateral gene transfer were identified using a combination of phylogenetic and BLAST-based analysis (see Additional file 9). Both *Pelobacter *species catabolize butanediol and acetoin [12]. The butanediol dehydrogenase (Bdh, Pcar_0330) and acetoin dehydrogenase (AcoABCL, Pcar_0343–Pcar0346), which catalyze the initial steps in the metabolism of these compounds, have been characterized in *P. carbinolicus *[32]. *P. propionicus *has genes with high sequence similarity to these, but the operon structure of the putative acetoin dehydrogenase included a duplication of the A and B subunits (Bdh, Ppro_1043 and AcoABCABL, Ppro_1024–Ppro1029, see Additional file 1). Phylogenetic analysis showed that the most closely related enzymes are not from *Geobacter *or *Desulfuromonas*, nor any other *delta*-*Proteobacteria *species (see Additional file 10). Instead the *Pelobacter *genes were most closely related to the butanediol catabolic genes from *Pseudomonas *[33] and Gram-positive species, indicating that these genes originated from lateral gene transfer (see Additional file 10).

The subsequent steps in the catabolism of the acetyl-CoA and acetaldehyde from acetoin are markedly different in the two *Pelobacter *species. The pathway in *P. propionicus *is cyclic and requires membrane-bound electron transport enzymes (Figure 3) [34]. Several of the key enzymes were predicted to have been acquired by lateral gene transfer. Pyruvate:ferredoxin oxidoreductase is required to convert acetyl-CoA to pyruvate (Figure 3), and there were two heterotetrameric pyruvate:ferredoxin oxidoreductases from lateral gene transfer. One (Ppro_0322–Ppro_0325, see Additional file 1) is most closely related to the enzymes from *Clostridia tetani *and *Thermotoga *species, and the other (Ppro_0469–Ppro_0472, see Additional file 1) is most similar to the enzyme in *Syntrophobacter *and several archaeal species.

Pyruvate is then converted to succinyl-CoA to regenerate NAD^+ ^without the need for a cooperating hydrogen-oxidizing species (Figure 3). *P. propionicus *has orthologs of four TCA cycle enzymes and the NADH dehydrogenase from the respiratory *Geobacteraceae *that could carry out these reactions (Figure 3, see Additional file 7). Then, propionate is generated via a methylmalonyl-CoA mutase and transcarboxylase (Figure 3). Both of these enzyme were predicted to have been acquired from lateral gene transfer. The mutase (Ppro_1284–Ppro_1285, see Additional file 1) is related to enzymes from *Chlorflexus *and *Geobacillus *species, and the transcarboxylase (Ppro_0033–Ppro_0034, see Additional file 1) is related to that crystallized from *Propionibacterium freudenreichii *[35].

Thus, it appears that *P. propionicus *uses a mosaic of vertically inherited and laterally acquired genes for fermentation. Interestingly, the pathway uses a complete, vertically inherited, respiratory electron transport chain – the succinate dehydrogenase operates in reverse as a fumarate reductase, accepting reducing equivalents from the NADH dehydrogenase [34]. This is equivalent to that used by *Geobacter *species growing by fumarate respiration [18,36].

### Differences between Pelobacter species associated with syntrophy

**Figure 4:**
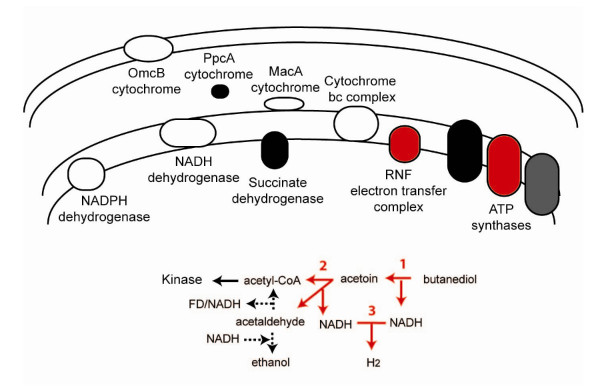
**The pathways of energy metabolism in *Pelobacter carbinolicus *and the differences from respiratory *Geobacteraceae *species**. Shown are the pathways of butanediol and acetoin ethanol fermentation and syntrophy used by *P. carbinolicus*. Vertically-inherited enzymes with orthologs in the other *Geobacteraceae *are shown in black. The vertically-inherited ATP synthase has been duplicated in *P. carbinolicus *and is shown in dark grey. Enzymes acquired by lateral gene transfer are shown in red: 1) butanediol dehydrogenase, 2) acetoin dehydrogenase, 3) hydrogenases, the RNF electron transfer complex, and possibly an ATP synthase (see text). Enzymes conserved in the respiratory *Geobacteraceae *but lost in *P. carbinolicus *are shown in white. Reactions with multiple enzymes encoded in the genome for which there are diverse conservation patterns are shown as dashed arrows.

*P. carbinolicus *ferments via much simpler pathway of cytoplasmic enzymes (Figure 4). As described above, the butanediol dehydrogenase and acetoin dehydrogenase appear to have been acquired by lateral gene transfer. NAD^+ ^is regenerated either by ethanol production or by proton reduction to hydrogen, which requires a syntrophic partner and ATP is made by substrate level phosphorylation by an acetate kinase [12].

Some *Pelobacter *species live in syntrophic associations with methanogens by partially oxidizing organic compounds to hydrogen and acetate which the methanogens consume [12,13]. Both *Pelobacter *species lacked orthologs to any of the *Geobacter*-type [37] hydrogenases (GSU0121–GSU0123, GSU0782–GSU0785, GSU2417–GSU2420 or GSU2718–GSU2722, see Additional file 1). Furthermore, the hydrogenases in the two *Pelobacter *species are dissimilar. This may reflect the fact that *P. carbinolicus *is capable of growing syntrophically with a hydrogen-consuming partner, most typically a methanogen [12]. Reducing equivalents can be disposed with the production of hydrogen, as long as the methanogen maintains hydrogen concentrations low enough for hydrogen production to be thermodynamically favorable. *P. propionicus *does not grow in this manner [12]. *P. carbinolicus *has two highly-similar, four-subunit hydrogenases (Pcar_1602–Pcar_1605 and Pcar_1633–Pcar_1636) related to the cytoplasmic, hydrogen-producing HndABCD hydrogenase in *Desulfovibrio fructosovorans *[38]. *P. propionicus*, encodes two highly-similar, 12-subunit enzymes (Ppro_0587–Ppro_0598 and Ppro_3521–Ppro_3532) that are related to the membrane-bound, hydrogen-producing, NiFe hydrogenase in *Pyrococcus furiosus *[39].

Approximately -45 kJ/mol is predicted to be available to *P. carbinolicus *and its methanogenic partner when they grow by ethanol oxidation under syntrophic conditions [13]. This is less than what is predicted to be required for generation of an ATP by substrate-level phosphorylation, and it is assumed that some of the ATP from acetyl-CoA conversion must be reinvested to drive the overall reaction, possibly by reverse electron transport [13]. It has been suggested that a Rnf-type ion-translocating electron transfer complex may play a role in reverse electron transport in syntrophs [40], by generating low potential ferredoxin from NADH using the membrane potential [41]. *P. carbinolicus *contains an Rnf gene cluster (Pcar_0260–Pcar_0265, Figure 4) that is similar to the enzymes from *Rhodobacter *and *Clostridium *species, both predicted to be involved in reduction of ferredoxin by NADH [42]. *P. propionicus*, which does not grow syntrophically, contains no Rnf-like genes.

Both *Pelobacter *genomes contain unusual numbers and types of ATP synthases, which have been suggested to be important for maintenance of the membrane potential [40]. Both contain the ATP synthase that is conserved in all of the *Geobacteraceae*, but these genes appear to have recently been duplicated in both organisms (Pcar_0015–Pcar_0016/Pcar_3130–Pcar_3136, Pcar_0944–Pcar_0952, and Ppro_0599–Ppro_0607, Ppro_1500–Ppro_1508, Figures 3 and 4). In addition, both *Pelobacter *species also have a third ATP synthase encoded in operons organized like that of *Methanosarcina barkeri *[43], though they were predicted to have been acquired from different sources. The genes in *P. propionicus *(Ppro_844–Ppro851) are most closely related to enzymes from species of *gamma Proteobacteria*: *Hahella*, *Legionella*, and *Methylococcus*. The *P. carbinolicus *genes (Pcar_2989–Pcar2997) are only distantly related to the *Geobacter *and *Desulfuromonas *operons, but because they are similar to genes from a variety of species including *D. acetoxidans*, it is difficult to be confident about the lateral transfer source.

## Conclusion

These results provide insights into the evolution of *Geobacteraceae *species into different environmental niches and biotechnological applications. The results suggest that the last common ancestor of the *Geobacteraceae *was an acetate-oxidizing, respiratory species capable of extracellular electron transfer, and that specialization for fermentative/syntrophic growth evolved at least twice, allowing some *Geobacteraceae *to fill additional niches.

The primacy of the respiratory mode is evident from the conservation of genes for all steps in this process including acetate uptake, central metabolism, and electron transfer across the cell membranes in both of the clades of the family. The fermentative/syntrophic *Pelobacter *species also contain many of these genes. However, they have lost several key enzymes that leave the pathways incomplete, including several necessary for the oxidation of acetate and most of the cytochromes predicted to provide the electrical connection between the inner membrane and the outside of the cell. Instead, the *Pelobacter *species have appropriated genes via lateral gene transfer for fermentative/syntrophic growth. It is clear that this has happened on two separate occasions. Although both *P. carbinolicus *and *P. propionicus *have closely related dehydrogenase genes for the initial metabolism of their unique substrates, acetoin and 2,3-butanediol, the genes for the further fermentation of these substrates are unrelated in the two organisms, reflecting the separate evolution of distinct metabolic pathways. The fact that *P. carbinolicus *also fills a syntrophic niche, participating in interspecies hydrogen transfer with hydrogen-consuming methanogens, whereas *P. propionicus *does not, may be explained by the genes associated with reverse electron transfer that only *P. carbinolicus *has appropriated. The fact that both *Pelobacter *species have phylogenetically distinct hydrogenase genes that are different from each other as well as those of the *Geobacter *species, may also reflect the difference in syntrophic capabilities of these species.

The selective pressure to specialize in syntrophic/fermentative growth may have initially been found at the interface of redox boundaries in sedimentary environments. As respiratory *Geobacteraceae *deplete the supply of the terminal electron acceptor Fe(III) oxide, their capacity for growth is greatly diminished and organisms with other respiratory processes, such as sulfate reduction or methane production, become predominant [2]. Some *Geobacter *species can oxidize acetate to carbon dioxide and hydrogen when they lack external electron acceptors [44], but the slow rate of this metabolism and the requirement for very low hydrogen partial pressures means that they are not competitive with acetate-utilizing sulfate reducers or methanogens. Acquiring the ability to ferment novel substrates and/or to grow syntrophically could have facilitated expansion into Fe(III) oxide depleted environments. Under such conditions investing energy in the production of respiratory enzymes such as the *c*-type cytochromes *Geobacteraceae *require to grow under Fe(III)-reducing conditions would be maladaptive.

These findings also provide insight into the types of metabolic changes that might take place as these organisms are being adapted for modern biotechnical applications. In applications such as the *in situ *bioremediation of uranium-contaminated groundwater and the conversion of organic compounds to electricity *Geobacter *species must deal with a scarcity of electron acceptor because electron donor is generally provided well in excess of electron acceptor availability. During *in situ *bioremediation Fe(III) oxides are rapidly depleted near the source of subsurface acetate amendments, limiting the growth and effectiveness of *Geobacter*-catalyzed U(VI) reduction [8]. *Geobacter *species form thick biofilms on the electrodes of microbial fuel cells, forcing many of the cells to metabolize acetate at a significant distance from this artificial electron acceptor [45,46]. Preliminary results suggest that, like the *Pelobacter *species described here, *Geobacteraceae *that predominate during *in situ *uranium reduction or on the anodes of microbial fuel cells have fewer *c*-type cytochromes (DRL, unpublished data). Enhanced ability to release excess electrons as hydrogen, in a manner similar to that of *P. carbinolicus*, could also be beneficial under conditions in which electron acceptor availability is limiting. Thus, these relatively few changes appear to have allowed a respiratory ancestor to radiate out into fermentative and syntrophic niches in addition to their respiratory roles in anaerobic environments. This information serves as a guide to the history of these organisms and provides information that could aid in optimizing their biotechnological applications.

## Methods

### Genome sequencing and annotation

With the exception of *G. sulfurreducens *[16], sequence data for the *Geobacteraceae *genomes were produced by the US Department of Energy Joint Genome Institute , using a whole-genome shotgun strategy for the Sanger-sequencing of 3-Kb, 8-Kb, and 40-Kb DNA libraries to 8-9X depth. Open reading frames and their translations and predicted function based on automated annotation were taken from NCBI  (Table 1).

### Clustering orthologs into protein families

All proteins in the genomes were clustered into families of orthologs and recent paralogs using OrthoMCL [17], which uses reciprocal best similarity pairs from all-vs-all BLAST [47] to identify orthologs and recent paralogs, which are then clustered together across all the genomes using the Markov clustering algorithm [48]. A functional role was predicted for each cluster using the *G. sulfurreducens in silico *model annotation [18] and COG categorization [19].

### Phylogenomics

All the ORFs from the six *Geobacteraceae *genomes and the outgroup species *Anaeromyxobacter dehalogenans *2CP-C (NC_007760) were put into orthologous groups using Hal [49], with inflation parameters from 1.1–5.0 for the clustering algorithm. The proteins used for the phylogeny were those that were part of a cluster generated with any inflation value that had exactly one member from each genome (see Additional file 6). All of the proteins in the cluster were concatenated and the resulting sequences aligned by ClustalW [50]. ProtTest [51] was used to select a model of molecular evolution and MrBayes [52] was used to create a Bayesian estimation of the phylogeny.

### Lateral gene transfer

A phylogenetic tree was inferred using PhyloGenie [53] for every protein from the six genomes. Homologous sequences for each protein were selected by BLAST from the non-redundant protein database from NCBI , alignments were created with ClustalW [50], and the phylogeny was inferred using neighbor-joining [54] and 100 bootstrapped replicates. These trees were used to identify proteins for which the nearest relative was not from the *Geobacteraceae*. If the phylogeny was strongly supported (bootstrap ≥ 50) or if the phylogeny was weakly supported and the most similar sequence in the non-redundant protein database from NCBI was not a *Geobacteraceae *species, the protein was considered a candidate. If the next branch out contained a single sequence not from *Geobacteraceae *species, the query gene was defined as being from lateral transfer. If the next branch contained a single sequence from *Geobacteraceae*, it was not. If the sister group was a clade or was not strongly supported, the ancestral condition was inferred [55] and used to determine lateral transfer.

## Authors' contributions

JEB carried out the conservation analysis, created the physiological models, constructed the single gene phylogenies, and drafted the manuscript. NDY designed the method for and carried out the lateral gene transfer prediction, constructed the whole genome phylogeny, and carried out the clustering method. DRL conceived of the study and helped draft the manuscript. All authors read and approved the final manuscript.

## Supplementary Material

Additional file 1 **All proteins referenced in this study. Spreadsheet with NCBI identification numbers and descriptions including name, predicted function, COG membership, size, location in genome, protein family, family size, family conservation pattern, lateral transfer prediction, and heme-binding motifs.** Click here for file

Additional file 2 **Protein families and their members from each of the genomes. Spreadsheet showing protein families of orthologs, with descriptions including member proteins, member genomes, family size, and conservation pattern.** Click here for file

Additional file 3 **Frequency of the phyletic patterns of protein conservation. **Spreadsheet showing the number of proteins with a given pattern of conservation. Click here for file

Additional file 4 **Proteins conserved only within the respiratory species.** Spreadsheet showing the proteins that were conserved in all and only the respiratory species. Click here for file

Additional file 5 **Proteins conserved only within the fermentative species.** Spreadsheet showing the proteins that were conserved in all and only the fermentative species. Click here for file

Additional file 6 **Proteins used in the whole genome phylogeny. **Spreadsheet showing the proteins used and their COG single-letter code and COG functional categorization. Click here for file

Additional file 7 **Conservation patterns for proteins involved in the energy metabolism of anaerobic respiration.** Spreadsheet showing all of the proteins with their metabolic role, conservation pattern, reaction abbreviation in the constraint-based model, and protein family. Click here for file

Additional file 8 **Phylogeny of the *Geobacteraceae *isocitrate dehydrogenases.** Figure showing a neighbor joining model of the phylogeny of these proteins with NCBI identification numbers. Click here for file

Additional file 9 **Genes predicted to have been acquired by lateral gene transfer.** Spreadsheet showing only those genes predicted to have been acquired by lateral gene transfer. Click here for file

Additional file 10 **Phylogeny of the *Pelobacter *butanediol dehydrogenases.** Figure showing a neighbor joining model of the phylogeny of these proteins with NCBI identification numbers. Click here for file

## References

[B1] Lovley DR, Holmes DE, Nevin KP (2004). Dissimilatory Fe(III) and Mn(IV) reduction. Adv Microb Physiol.

[B2] Lovley DR (1991). Dissimilatory Fe(III) and Mn(IV) reduction. Microbiol Rev.

[B3] Lovley DR (2003). Cleaning up with genomics: applying molecular biology to bioremediation. Nat Rev Microbiol.

[B4] Lovley DR, Nevin KP, Wall J (2008). Electricity production with electricigens. Bioenergy.

[B5] Lovley DR, Coates JD, Blunt-Harris EL, Phillips EJ, Woodward JC (1996). Humic substances as electron acceptors for microbial respiration. Nature.

[B6] Lovley DR, Woodward JC, Chapelle FH (1994). Stimulated anoxic biodegradation of aromatic hydrocarbons using Fe(III) ligands. Nature.

[B7] Lovley DR, Phillips EJ, Gorby YA, Landa ER (1991). Microbial reduction of uranium. Nature.

[B8] Anderson RT, Vrionis HA, Ortiz-Bernad I, Resch CT, Long PE, Dayvault R, Karp K, Marutzky S, Metzler DR, Peacock A, White DC, Lowe M, Lovley DR (2003). Stimulating the in situ activity of *Geobacter *species to remove uranium from the groundwater of a uranium-contaminated aquifer. Appl Environ Microbiol.

[B9] Bond DR, Holmes DE, Tender LM, Lovley DR (2002). Electrode-reducing microorganisms that harvest energy from marine sediments. Science.

[B10] Lovley DR (2006). Bug juice: harvesting electricity with microorganisms. Nat Rev Microbiol.

[B11] Tender LM, Reimers CE, Stecher HA, Holmes DE, Bond DR, Lowy DA, Pilobello K, Fertig SJ, Lovley DR (2002). Harnessing microbially generated power on the seafloor. Nat Biotechnol.

[B12] Schink B (1984). Fermentation of 2,3-butanediol by *Pelobacter carbinolicus sp. nov. *and *Pelobacter propionicus sp. nov.*, and evidence for propionate formation from C-2 compounds. Arch Microbiol.

[B13] Schink B (1997). Energetics of syntrophic cooperation in methanogenic degradation. Microbiol Mol Biol Rev.

[B14] Haveman SA, Didonato RJ, Villanueva L, Shelobolina ES, Postier B, Xu A, Liu A, Lovley DR (2008). Genome-Wide Gene Expression Patterns and Growth Requirements Suggest that *Pelobacter carbinolicus *Reduces Fe(III) Indirectly via Sulfide Production. Appl Environ Microbiol.

[B15] Richter H, Lanthier M, Nevin KP, Lovley DR (2007). Lack of electricity production by *Pelobacter carbinolicus *indicates that the capacity for Fe(III) oxide reduction does not necessarily confer electron transfer ability to fuel cell anodes. Appl Environ Microbiol.

[B16] Methe BA, Nelson KE, Eisen JA, Paulsen IT, Nelson W, Heidelberg JF, Wu D, Wu M, Ward N, Beanan MJ, Dodson RJ, Madupu R, Brinkac LM, Daugherty SC, DeBoy RT, Durkin AS, Gwinn M, Kolonay JF, Sullivan SA, Haft DH, Selengut J, Davidsen TM, Zafar N, White O, Tran B, Romero C, Forberger HA, Weidman J, Khouri H, Feldblyum TV (2003). Genome of *Geobacter sulfurreducens*: metal reduction in subsurface environments. Science.

[B17] Li L, Stoeckert CJ, Roos DS (2003). OrthoMCL: identification of ortholog groups for eukaryotic genomes. Genome Res.

[B18] Mahadevan R, Bond DR, Butler JE, Esteve-Nunez A, Coppi MV, Palsson BO, Schilling CH, Lovley DR (2006). Characterization of metabolism in the Fe(III)-reducing organism *Geobacter sulfurreducens *by constraint-based modeling. Appl Environ Microbiol.

[B19] Tatusov RL, Koonin EV, Lipman DJ (1997). A genomic perspective on protein families. Science.

[B20] Holmes DE, Nevin KP, Lovley DR (2004). Comparison of 16S rRNA, nifD, recA, gyrB, rpoB and fusA genes within the family *Geobacteraceae fam. nov*. Int J Syst Evol Microbiol.

[B21] Champine JE, Goodwin S (1991). Acetate catabolism in the dissimilatory iron-reducing isolate GS-15. J Bacteriol.

[B22] Galushko AS, Schink B (2000). Oxidation of acetate through reactions of the citric acid cycle by *Geobacter sulfurreducens *in pure culture and in syntrophic coculture. Arch Microbiol.

[B23] Paulsen J, Kroger A, Thauer RK (1986). ATP-driven succinate oxidation in the catabolism of *Desulfuromonas acetoxidans*. Arch Microbiol.

[B24] Risso C, Methe BA, Elifantz H, Holmes DE, Lovley DR (2008). Highly Conserved Genes in *Geobacter *Species with Expression Patterns Indicative of Acetate Limitation. Microbiology.

[B25] Imabayashi F, Aich S, Prasad L, Delbaere LT (2006). Substrate-free structure of a monomeric NADP isocitrate dehydrogenase: an open conformation phylogenetic relationship of isocitrate dehydrogenase. Proteins.

[B26] Coppi MV, O'Neil RA, Leang C, Kaufmann F, Methe BA, Nevin KP, Woodard TL, Liu A, Lovley DR (2007). Involvement of *Geobacter sulfurreducens *SfrAB in acetate metabolism rather than intracellular, respiration-linked Fe(III)-citrate reduction. Microbiology.

[B27] Tseng CP, Yu CC, Lin HH, Chang CY, Kuo JT (2001). Oxygen- and growth rate-dependent regulation of *Escherichia coli *fumarase (FumA, FumB, and FumC) activity. J Bacteriol.

[B28] Schutz M, Brugna M, Lebrun E, Baymann F, Huber R, Stetter KO, Hauska G, Toci R, Lemesle-Meunier D, Tron P, Schmidt C, Nitschke W (2000). Early evolution of cytochrome bc complexes. J Mol Biol.

[B29] Allen JW, Daltrop O, Stevens JM, Ferguson SJ (2003). C-type cytochromes: diverse structures and biogenesis systems pose evolutionary problems. Philos Trans R Soc Lond B Biol Sci.

[B30] Butler JE, Kaufmann F, Coppi MV, Nunez C, Lovley DR (2004). MacA, a diheme c-type cytochrome involved in Fe(III) reduction by *Geobacter sulfurreducens*. J Bacteriol.

[B31] Leang C, Coppi MV, Lovley DR (2003). OmcB, a *c*-type polyheme cytochrome, involved in Fe(III) reduction in *Geobacter sulfurreducens*. J Bacteriol.

[B32] Oppermann FB, Steinbuchel A (1994). Identification and molecular characterization of the aco genes encoding the *Pelobacter carbinolicus *acetoin dehydrogenase enzyme system. J Bacteriol.

[B33] Huang M, Oppermann FB, Steinbuchel A (1994). Molecular characterization of the *Pseudomonas putida *2,3-butanediol catabolic pathway. FEMS Microbiol Lett.

[B34] Schink B, Kremer DR, Hansen TA (1987). Pathway of propionate formation from ethanol in *Pelobacter propionicus*. Arch Microbiol.

[B35] Reddy DV, Shenoy BC, Carey PR, Sonnichsen FD (2000). High resolution solution structure of the 1.3S subunit of transcarboxylase from *Propionibacterium shermanii*. Biochemistry.

[B36] Butler JE, Glaven RH, Esteve-Nunez A, Nunez C, Shelobolina ES, Bond DR, Lovley DR (2006). Genetic characterization of a single bifunctional enzyme for fumarate reduction and succinate oxidation in *Geobacter sulfurreducens *and engineering of fumarate reduction in *Geobacter metallireducens*. J Bacteriol.

[B37] Coppi MV (2005). The hydrogenases of *Geobacter sulfurreducens*: a comparative genomic perspective. Microbiology.

[B38] Malki S, Saimmaime I, De Luca G, Rousset M, Dermoun Z, Belaich JP (1995). Characterization of an operon encoding an NADP-reducing hydrogenase in *Desulfovibrio fructosovorans*. J Bacteriol.

[B39] Sapra R, Verhagen MF, Adams MW (2000). Purification and characterization of a membrane-bound hydrogenase from the hyperthermophilic archaeon *Pyrococcus furiosus*. J Bacteriol.

[B40] McInerney MJ, Rohlin L, Mouttaki H, Kim U, Krupp RS, Rios-Hernandez L, Sieber J, Struchtemeyer CG, Bhattacharyya A, Campbell JW, Gunsalus RP (2007). The genome of *Syntrophus aciditrophicus*: life at the thermodynamic limit of microbial growth. Proc Natl Acad Sci USA.

[B41] Kumagai H, Fujiwara T, Matsubara H, Saeki K (1997). Membrane localization, topology, and mutual stabilization of the rnfABC gene products in *Rhodobacter capsulatus *and implications for a new family of energy-coupling NADH oxidoreductases. Biochemistry.

[B42] Kim J, Hetzel M, Boiangiu CD, Buckel W (2004). Dehydration of (R)-2-hydroxyacyl-CoA to enoyl-CoA in the fermentation of alpha-amino acids by anaerobic bacteria. FEMS Microbiol Rev.

[B43] Sumi M, Yohda M, Koga Y, Yoshida M (1997). F0F1-ATPase genes from an archaebacterium, *Methanosarcina barkeri*. Biochem Biophys Res Commun.

[B44] Cord-Ruwisch R, Lovley DR, Schink B (1998). Growth of *Geobacter sulfurreducens *with acetate in syntrophic cooperation with hydrogen-oxidizing anaerobic partners. Appl Environ Microbiol.

[B45] Nevin KP, Richter H, Covalla SF, Johnson JP, Woodard TL, Orloff AL, Jia H, Zhang M, Lovley DR (2008). Power output and columbic efficiencies from biofilms of *Geobacter sulfurreducens *comparable to mixed community microbial fuel cells. Environ Microbiol.

[B46] Reguera G, Nevin KP, Nicoll JS, Covalla SF, Woodard TL, Lovley DR (2006). Biofilm and nanowire production leads to increased current in *Geobacter sulfurreducens *fuel cells. Appl Environ Microbiol.

[B47] Altschul SF, Madden TL, Schaffer AA, Zhang J, Zhang Z, Miller W, Lipman DJ (1997). Gapped BLAST and PSI-BLAST: a new generation of protein database search programs. Nucleic Acids Res.

[B48] Enright AJ, Van Dongen S, Ouzounis CA (2002). An efficient algorithm for large-scale detection of protein families. Nucleic Acids Res.

[B49] Robbertse B, Reeves JB, Schoch CL, Spatafora JW (2006). A phylogenomic analysis of the *Ascomycota*. Fungal Genet Biol.

[B50] Thompson JD, Higgins DG, Gibson TJ (1994). CLUSTAL W: improving the sensitivity of progressive multiple sequence alignment through sequence weighting, position-specific gap penalties and weight matrix choice. Nucleic Acids Res.

[B51] Abascal F, Zardoya R, Posada D (2005). ProtTest: selection of best-fit models of protein evolution. Bioinformatics.

[B52] Ronquist F, Huelsenbeck JP (2003). MrBayes 3: Bayesian phylogenetic inference under mixed models. Bioinformatics.

[B53] Frickey T, Lupas AN (2004). PhyloGenie: automated phylome generation and analysis. Nucleic Acids Res.

[B54] Saitou N, Nei M (1987). The neighbor-joining method: a new method for reconstructing phylogenetic trees. Mol Biol Evol.

[B55] Swofford DL, Madison WP (1987). Reconstructing ancestral character states under Wagner parsimony. Math Biosci.

