# Supplementary material for: Evolution from a respiratory ancestor to fill syntrophic and fermentative niches: comparative fenomics of six Geobacteraceae species
Source: BMC Genomics. 2009 Mar 11;10:103. doi: 10.1186/1471-2164-10-103 (PMC2669807; doi:10.1186/1471-2164-10-103)
Supplement: Additional file 8 — Phylogeny of the Geobacteraceae isocitrate dehydrogenases. Figure showing a neighbor joining model of the phylogeny of these proteins with NCBI identification numbers. [file 1471-2164-10-103-S8.ppt]

## Slide 1
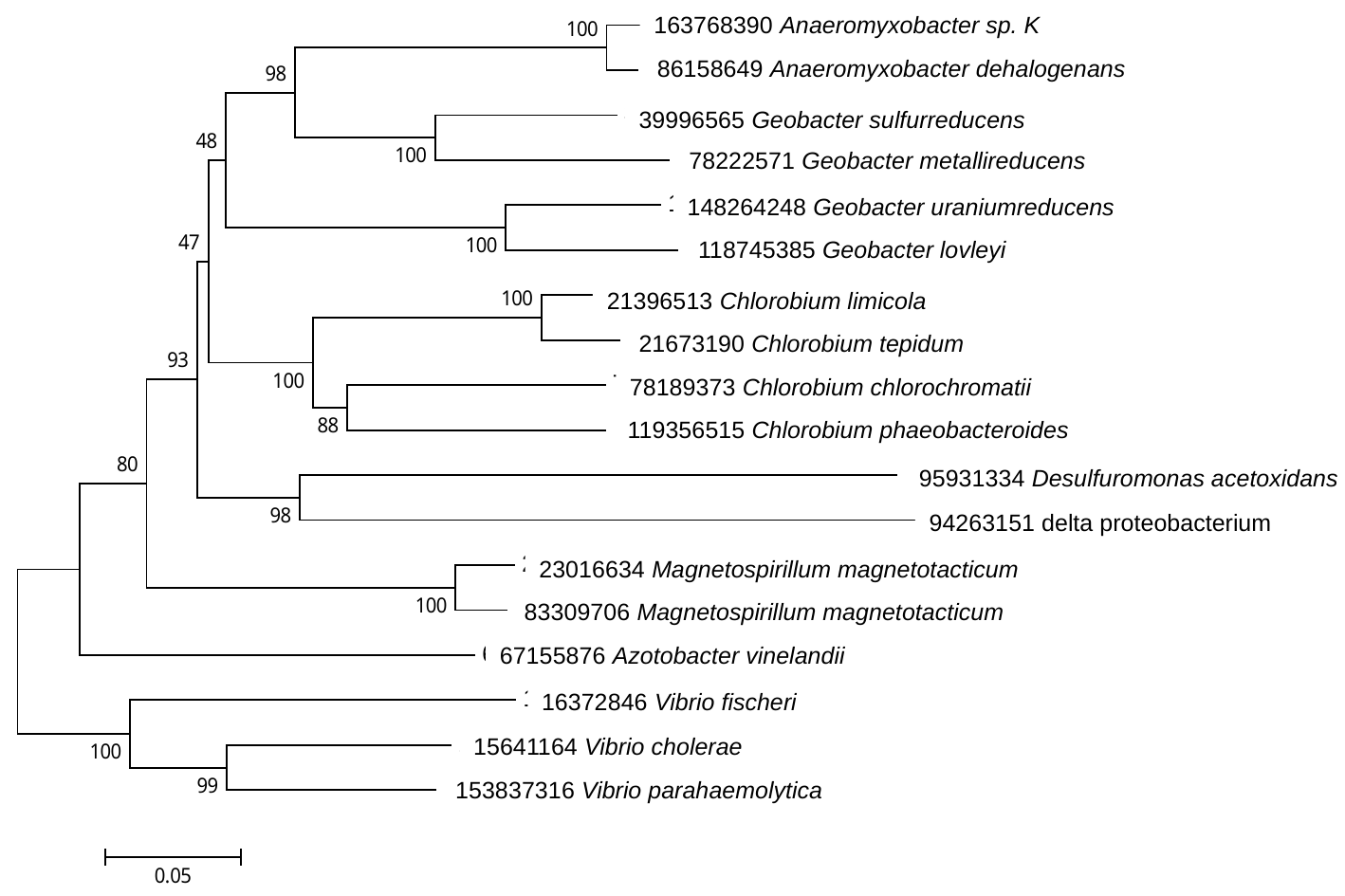

163768390 Anaeromyxobacter sp. K
86158649 Anaeromyxobacter dehalogenans
39996565 Geobacter sulfurreducens
78222571 Geobacter metallireducens
148264248 Geobacter uraniumreducens
118745385 Geobacter lovleyi
21396513 Chlorobium limicola
21673190 Chlorobium tepidum
78189373 Chlorobium chlorochromatii
119356515 Chlorobium phaeobacteroides
95931334 Desulfuromonas acetoxidans
94263151 delta proteobacterium
23016634 Magnetospirillum magnetotacticum
83309706 Magnetospirillum magnetotacticum
67155876 Azotobacter vinelandii
16372846 Vibrio fischeri
15641164 Vibrio cholerae
153837316 Vibrio parahaemolytica
