# Supplementary figures and images for: Evolution from a respiratory ancestor to fill syntrophic and fermentative niches: comparative fenomics of six Geobacteraceae species
Source: BMC Genomics. 2009 Mar 11;10:103. doi: 10.1186/1471-2164-10-103 (PMC2669807; doi:10.1186/1471-2164-10-103)

## Slide 1
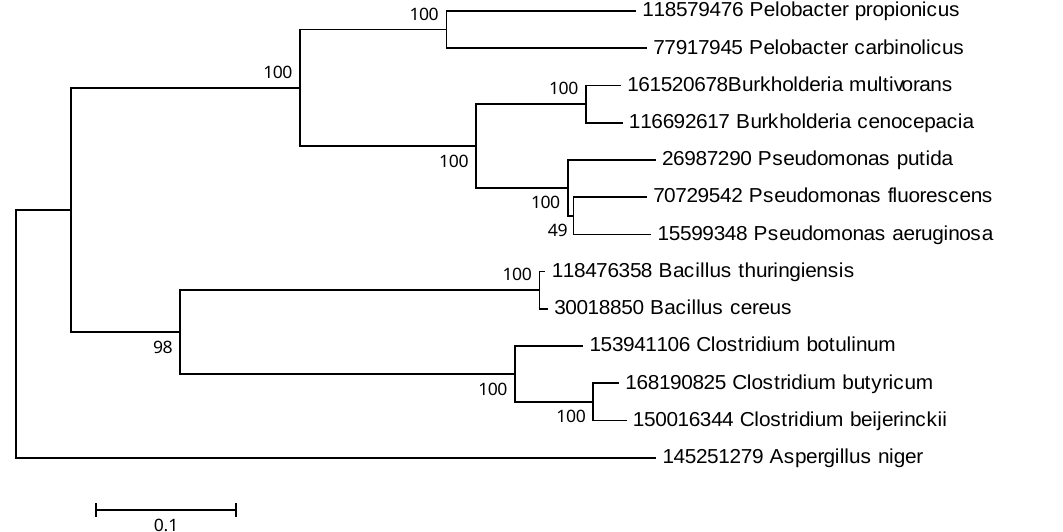

Supplement: Additional file 10 — Phylogeny of the Pelobacter butanediol dehydrogenases. Figure showing a neighbor joining model of the phylogeny of these proteins with NCBI identification numbers. [file 1471-2164-10-103-S10.ppt]
